# Cryo-FIB–enabled 3D imaging of biological specimens

**DOI:** 10.1186/s42649-026-00131-z

**Published:** 2026-04-17

**Authors:** Seonhye Son, Sujin Lee, Jihyun Kim, Chihong Song

**Affiliations:** 1 Core Research Facility, Pusan National University, Yangsan, 50612 Republic of Korea; 2 Department of Convergence Medicine, School of Medicine, Pusan National University, Yangsan, 50612 Republic of Korea; 3 Medical Research Institute, Pusan National University School of Medicine, Yangsan, 50612 Republic of Korea

**Keywords:** Cryo-FIB-SEM, cryo-electron tomography, cryo-correlative light and electron microscopy (cryo-CLEM), high-pressure freezing, slice-and-view

## Abstract

Cryo-electron tomography (cryo-ET) has become a key technique for observing the three-dimensional structures of biomolecular complexes and organelles in cellular and tissue environments in situ. However, for thicker specimens that cannot be directly imaged at an electron-transparent thickness, the practical success of cryo-ET depends largely on workflow design that reproducibly yields high-quality, target-containing lamellae. Cryo-FIB-SEM is the de facto standard platform for precisely machining frozen specimens to an electron-transparent thickness, and it integrates the overall process into a single system, encompassing targeting via cryo-CLEM, the management of major failure modes such as contamination, charging, curtaining, and downstream steps for data reconstruction and interpretation. This review summarizes a cryo-FIB-SEM–centered cryo-ET workflow from the perspectives of specimen preparation, targeting, milling, acquisition, reconstruction, and interpretation. In addition, we discuss extended preparation and imaging strategies for thicker specimens, including high-pressure freezing (HPF), the waffle method, cryo-FIB-SEM slice-and-view imaging, and Serial Lift-Out, highlighting both their expanding capabilities and the operational challenges that remain for robust and scalable implementation.

## Introduction

**Fig. 1:**
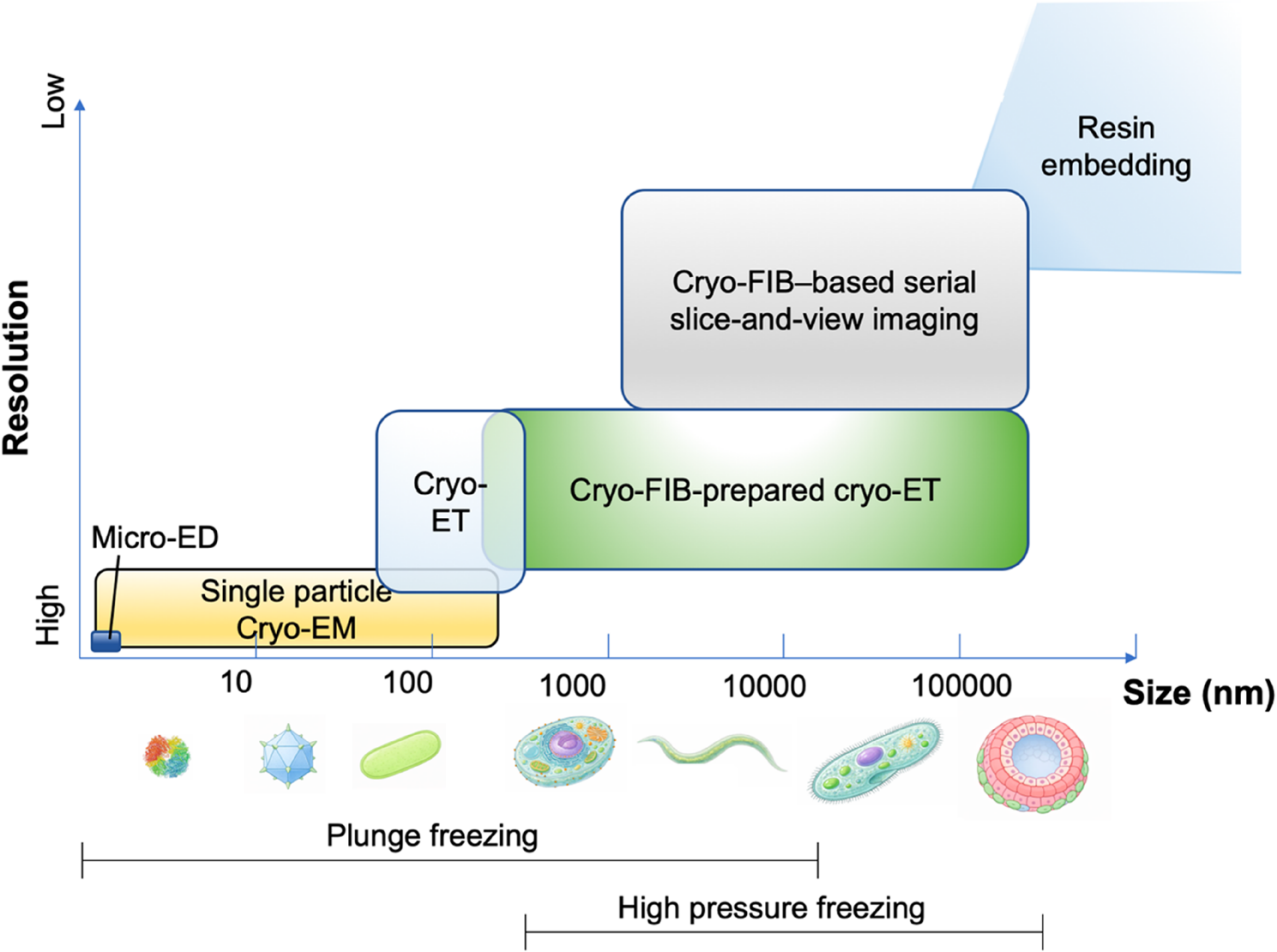
Multiscale applicability of cryo-EM modalities. Representative cryo-EM approaches are schematized as a function of specimen size (nm, x-axis) and achievable resolution (y-axis). MicroED and single-particle cryo-EM cover high-resolution regimes for relatively small specimens, whereas Cryo-FIB-independent Cryo-ET and Cryo-FIB-dependent Cryo-ET extend in situ 3D imaging to larger cellular volumes. Cryo-FIB–based serial slice-and-view imaging enables volumetric imaging at larger length scales, and resin embedding represents an alternative route for the largest specimens. The approximate applicable ranges of plunge freezing and high-pressure freezing are also indicated

**Fig. 2:**
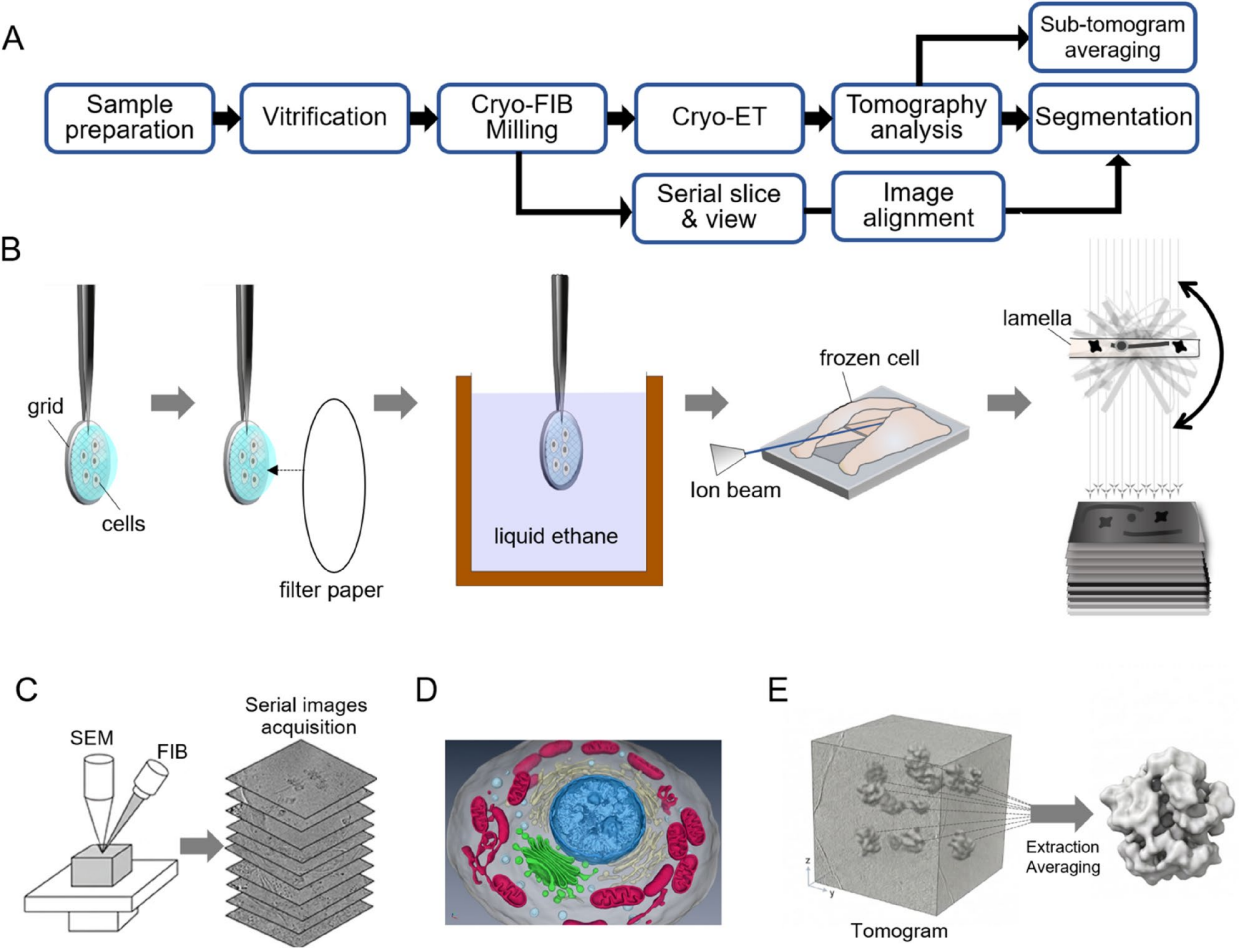
Overview of a cryo-FIB–based cryo-ET workflow.**A** Multiscale workflow enabled by cryo-FIB-SEM. The core process begins with sample preparation and vitrification, followed by cryo-FIB milling, cryo-ET data acquisition, tomographic reconstruction, and segmentation. In addition, sub-tomogram averaging can be performed after tomographic analysis to improve resolution by averaging repeated particles within tomograms. Large-area 3D volume imaging can be achieved using slice-and-view (serial block-face imaging). **B** Stepwise schematic of grid-based vitrification (blotting and plunge freezing into liquid ethane), cryo-FIB milling to generate an electron-transparent lamella from a frozen cell, and tilt-series acquisition for tomographic reconstruction. **C** Schematic illustration of serial slice-and-view imaging using cryo-FIB-SEM, in which sequential milling and SEM imaging are repeated to acquire serial images for large-volume 3D reconstruction. **D** Representative segmentation output showing the three-dimensional organization of intracellular structures reconstructed from tomographic or serial imaging data. **E** Schematic representation of sub-tomogram averaging, in which repeated macromolecular particles are extracted from a tomogram, aligned, classified, and averaged to improve structural resolution

Cryo-electron microscopy (cryo-EM) is a structural biology platform that preserves specimens in a near-hydrated state through vitrification by plunge freezing or high-pressure freezing, and enables multiscale imaging within a single framework, ranging from MicroED and single-particle analysis to cellular- and tissue-level imaging (Fig. 1). Within this multiscale spectrum, cryo-electron tomography (cryo-ET) provides direct three-dimensional structural information in situ and is currently one of the most actively developing areas. A major advantage of cryo-ET is that it can directly address the three-dimensional context of cells, including the location of molecular machines and their surrounding environment, beyond the averaged structures of purified protein complexes (Schur 2019). Nevertheless, cryo-ET projects remain technically demanding, not only because of instrument access, but also because reliably securing lamellae suitable for analysis requires a series of complex decisions. If a lamella is too thick, the signal-to-noise ratio decreases and multiple scattering increases. If it is too thin or nonuniform, structural integrity or target retention becomes difficult (Marko et al. 2007). More importantly, even a well-prepared lamella is of little use if it does not contain the target. Therefore, the bottleneck in cryo-ET is not the thinning technique itself, but the establishment of a workflow that produces high-quality, target-containing lamellae at high yield. From this perspective, cryo-focused ion beam-scanning electron microscopy (cryo-FIB-SEM) is not peripheral equipment for cryo-ET but rather occupies the center of the workflow (Fig. 2). Cryo-FIB-SEM not only allows frozen specimens to be machined to an electron-transparent thickness but also enables assessment of surface geometry by scanning electron microscopy (SEM), reduces targeting uncertainty by integrating cryogenic correlative light and electron microscopy (cryo-CLEM) when needed, and allows repeated verification at intermediate milling stages (Schorb and Briggs 2014; Gorelick et al. 2019). In other words, cryo-FIB-SEM is both a lamella fabrication instrument and the central platform for targeting, machining, and quality control (QC), which together determine cryo-ET yield (Fig. 2).

## Vitrification

One practical way to increase the success rate of cryo-ET is to design specimens with lamella fabrication in mind from the freezing step onward. Plunge freezing is the most widely used standard method and is sufficiently effective for specimens such as cultured cell monolayers or thin microbial samples (Figs. 1 and 2B ). However, when specimen thickness exceeds approximately 10 μm, plunge freezing often fails to provide sufficient heat transfer to the interior, thereby increasing the likelihood of vitrification failure. In addition, the positioning of the specimen on the grid (geometric arrangement) strongly influences subsequent millability and target accessibility. Especially for in situ cryo-ET, rather than aiming solely for perfectly thin and uniform ice, ensuring that a target-containing region is stably presented for subsequent milling may be more critical.

For thick specimens, high-pressure freezing (HPF) becomes a practical option (Fig. 1). By applying high pressure during freezing, HPF alters the phase behavior of water in a way that suppresses ice crystal nucleation and growth, thereby increasing the achievable vitrified depth in thicker samples. It therefore serves as a starting point for connecting specimens such as tissues, high-density pellets, and multicellular organisms to cryo-FIB–based workflows (Schaffer et al. 2019). In HPF-based approaches, the primary challenge shifts from achieving vitrification to locating the target and designing appropriate milling strategies. In thick specimens, landmarks are often unclear under SEM alone, and artifacts such as curtaining, charging, and contamination can occur more frequently during milling, making targeting and QC relatively more important. The ‘waffle method’ is an HPF-based workflow variant for thick specimens, in which a vitrified sample is first formed as a relatively thick slab on the grid and subsequently processed into lamellae by cryo-FIB machining. This strategy can improve throughput and success rates by reconfiguring thick specimens into a form more suitable for lamella fabrication (Kelley et al. 2022).

## Cryo-CLEM

**Fig. 3:**
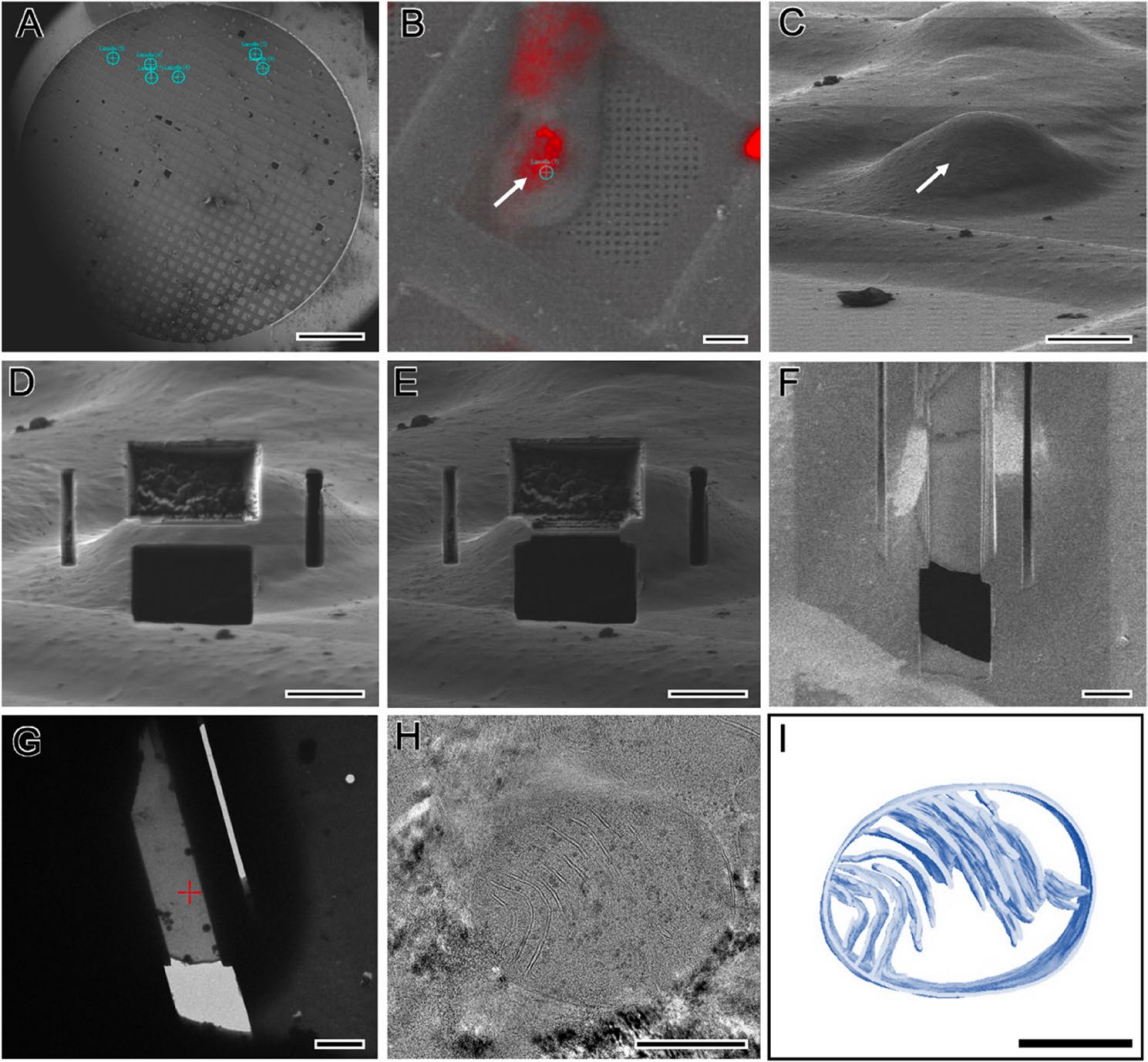
Representative example of cryo-FIB lamella preparation and the cryo-ET and segmentation workflow. **A** SEM overview image of a vitrified EM grid with candidate target sites indicated. **B** Example of fluorescence signals overlaid on the electron/SEM image to define a ROI and guide milling. Arrow indicates the position of mitochondria based on the fluorescence signal. **C** SEM image of the selected cell/region prior to milling. The arrow indicates the same position as in (**B**), with the stage tilted to the appropriate angle for FIB milling. **D**–**E** Trenching and progressive milling from coarse to fine to form a lamella at the target site. **F** Final lamella geometry suitable for subsequent cryo-ET analysis. **G** Overview of the lamella after transfer to the TEM. **H** Representative tomographic image obtained from the lamella, showing mitochondrial ultrastructure. **I** Example of 3D visualization based on segmentation of mitochondria. Scale bars: (A), 500 μm; (**B**–**C**), 10 μm; (**D**–**G**), 3 μm; (**H**,**I**), 500 nm

CLEM has evolved as an important approach for bridging the technical gap between light microscopy and electron microscopy. By combining the molecularly specific positional information and relatively wide-field target-detection capability provided by fluorescence-based light microscopy with the high-resolution ultrastructural information and precise morphological analysis offered by electron microscopy, it enables integrated interpretation of the location of a specific target together with its surrounding structural context. This complementarity is particularly important in cryo-ET, because the interior of cells consists of a complex structural background and diverse electron-dense components, making it often difficult to directly identify the position of a specific protein or target by cryo-ET alone. Cryo-CLEM enables the spatial localization of targets through fluorescent labeling or intrinsic signals, thereby allowing more accurate targeting for subsequent cryo-FIB milling and cryo-ET analysis (Fig. 3A-C).

One major failure mode in cryo-ET of milled specimens is that the lamella is well prepared, but the target is absent. This does not simply reflect bad luck; rather, it indicates that the positional information obtained from cryo-CLEM was not translated into the milling coordinates with sufficient accuracy. In this sense, the practical value of cryo-CLEM lies not merely in overlaying fluorescence and electron microscopy images, but in guiding where and how the specimen should be milled so that the target is retained within the final lamella. Because cryo-CLEM is ultimately a coordinate-registration problem, reliable targeting depends on how accurately positional information can be transferred across imaging modalities (Schorb and Briggs 2014). In practice, errors can arise from multiple sources, including distortion during coordinate transformation, differences in magnification and specimen orientation between LM and SEM/FIB, fiducial distribution, and morphological changes that occur during milling. Although each individual deviation may appear small, their cumulative effect can be decisive when the final lamella thickness is limited to merely a few hundred nanometers. Therefore, successful cryo-CLEM requires not only image correlation itself, but also repeated verification that the predicted target position remains consistent before and during milling, as well as a realistic evaluation of whether the ROI is likely to be retained within the final lamella volume. When such positional uncertainty is actively managed, cryo-CLEM becomes not merely image decoration, but a critical workflow component that provides reproducible yield even for rare targets (Gorelick et al. 2019; Yang et al. 2026).

## Cryo-FIB-SEM lamella fabrication

Cryo-FIB-SEM-based lamella fabrication generally proceeds in the order of protective layer deposition, trenching and rough milling, fine milling, and transfer to cryo-TEM **(**Figs. 3D-G). While this process is often framed in terms of how thin a lamella can be made, a more practical question from the cryo-ET perspective is how reliably a uniform region containing the target can be created. Thus, it is helpful to redefine the stepwise objectives in terms of quality and yield (Rigort and Plitzko 2015). The protective-layer step is intended mainly to reduce curtaining and charging and to mitigate surface damage. In thick or heterogeneous specimens, the choice of protective layer and deposition conditions can strongly influence subsequent milling outcomes. Rough milling and trenching determine the geometry of the lamella (Fig. 3D). The key at this stage is to safely secure a volume likely to contain the target while leaving sufficient margin for fine milling to create a uniform region. Fine milling reaches the final thickness, but in practice, uniformity, flatness, target retention, and minimization of contamination and charging are more important than thickness alone (Fig. 3E-F). Failure modes that frequently occur in practice are relatively well documented. Contamination tends to accumulate over time, making the number of transfers, exposure time, and chamber-condition management important. Charging directly harms contrast, focus, and drift and must be mitigated through coating, grounding, and optimization of conditions. Curtaining arises from material heterogeneity, insufficient coating, or inappropriate milling conditions, thereby degrading surface quality and reducing interpretability. Finally, devitrification can lead to irreversible damage, so the management of temperature, vacuum, and transfer time is a basic prerequisite. In this way, cryo-FIB-SEM-based lamella fabrication is not a single technique, but an operational problem of systematically reducing the probability of failure modes; yield improves when it is understood in this framework.

## Cryo-ET acquisition and reconstruction/STA

Once lamellae are secured, cryo-ET proceeds through tilt-series acquisition, alignment, reconstruction, contrast transfer function (CTF) correction, and, when needed, subtomogram averaging (STA) (Figs. 2 and 3H-I). At this stage, acquisition-level decisions play a critical role in determining downstream data quality and interpretability. For example, dose-symmetric tilt schemes are often preferred because they distribute the electron dose more evenly across the tilt series and help preserve high-resolution information. In addition, careful management of the total cumulative dose is essential, particularly for thin lamellae, where radiation damage can rapidly degrade structural integrity and image contrast. The accessible tilt range is also frequently constrained by lamella geometry, including thickness, orientation, and shadowing effects, which in turn influences angular coverage and reconstruction fidelity. Therefore, acquisition should be considered not merely as a routine step, but as an integral part of experimental design that directly impacts reconstruction quality.

An advantage here is that the standard toolchain is relatively well established. The IMOD/Etomo family remains widely used for alignment and reconstruction (Kremer et al. 1996), and tools that accelerate marker-free approaches are also routinely used (Zheng et al. 2022). STA can be performed in several software ecosystems, such as RELION tomography/STA, Dynamo, and emClarity, and the optimal choice differs depending on the study objective (Zivanov et al. 2022; Castaño-Díez et al. 2012; Himes and Zhang 2018).

A rapidly expanding recent trend is deep learning-based correction, restoration, and detection. Approaches that reduce anisotropy caused by the missing wedge or generalize particle detection within tomograms are actively being developed, thereby improving throughput and accessibility in practice (Buchholz et al. 2019; Liu et al. 2022; Wiedemann and Heckel 2024; Rice et al. 2023; Moebel et al. 2021). However, because such methods can generate plausible yet erroneous results, it is important to embed validation mechanisms into the workflow.

## Cryo-FIB-SEM slice-and-view imaging

As another capability provided by cryo-FIB-SEM, serial slice-and-view (serial block-face imaging) can be used as a complementary approach under certain conditions (Fig. 2C). Slice-and-view repeatedly removes thin layers and images the exposed surface by SEM to build a three-dimensional volume over a relatively wide field of view. This can provide broader cellular- and organelle-level context for the local high-resolution information provided by cryo-ET. However, because dataset size is large and the interpretive bottleneck tends to shift to segmentation, adopting slice-and-view requires clearly defined QC criteria from the acquisition stage to produce segmentation-ready data, including the management of charging, contamination, and surface damage, as well as stable slice thickness and metadata tracking. Therefore, due to the remaining technical challenges, slice-and-view is best regarded not as an essential element, but rather as an optional approach to consider when one wishes to augment contextual information in a cryo-ET project. For three-dimensional reconstruction of large-area samples, HPF followed by freeze-substitution or, for even larger specimens, resin embedding after conventional chemical fixation can provide more stable and reliable results (Fig. 1).

## Serial lift-out

**Fig. 4:**
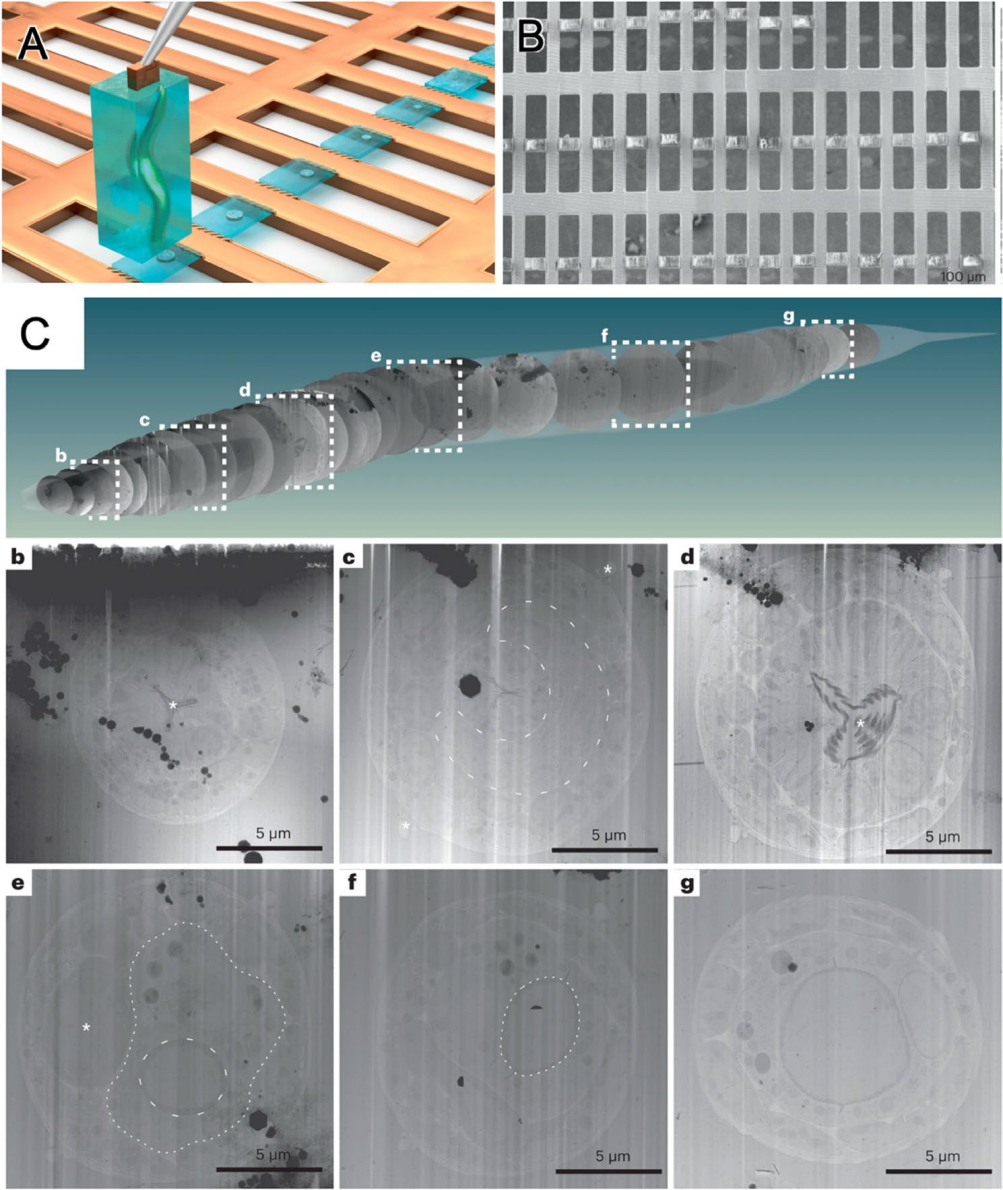
Serial lift-out workflow to expand sampling throughput and spatial context. **A** Schematic illustrating extraction of a large vitrified volume by lift-out, attachment to a receiver grid, and serial sectioning to generate multiple lamellae. **B** Example of a receiver grid populated with multiple serial lamellae (scale bar, 100 μm). **C** Example of tracking serial lamella positions along a whole-organism axis (dashed boxes), with representative lamella overview images (b–g) shown below. Adapted from figures of Schiøtz et al., 2024, Nature Methods (10.1038/s41592-023-02113-5)

In thick specimens, conventional on-grid lamella preparation places substantial constraints on cryo-ET observation, as each lamella provides access to only a thin and spatially restricted region of the specimen. Moreover, direct on-grid thinning of thick material requires extensive milling to remove large amounts of surrounding material, making the process time-consuming and increasing the likelihood of lamella damage or failure during preparation. These limitations can compromise both efficient target recovery and preservation of broader structural context. To address this problem, lift-out strategies were developed to extract a larger specimen volume and relocate it onto a receiver grid for subsequent thinning. Serial Lift-Out further extends this concept by serially sectioning a single extracted volume into multiple lamellae, thereby expanding sampling coverage and enabling more continuous three-dimensional reconstruction across a larger specimen axis. The application of Serial Lift-Out for the production of serial lamellae is exemplified in the study of C. elegans by (Schiøtz et al. 2024), as shown in Fig. 4. The workflow begins with the precise attachment of an extracted specimen volume onto a specialized receiver grid (Fig. 4A). This setup enables the production of multiple lamellae through serial sectioning, which are then arranged sequentially along the organism’s body axis (Fig. 4B). As shown in Fig. 4C, these lamellae provide high-resolution internal structural details, serving as high-quality substrates for cryo-ET data acquisition and sophisticated downstream analyses, such as subtomogram averaging. Taken together, these features highlight the potential of Serial Lift-Out as a specimen-preparation strategy for extending cryo-ET observation sites in thick specimens from a single lamella to multiple serial lamellae (Schiøtz et al. 2024). From a practical standpoint, however, successful implementation also depends on several operational factors, including the management of redeposition during milling, the mechanical stability of serial lamellae during transfer and imaging, and the balance between increased sampling coverage and preparation complexity. Compared with conventional on-grid milling, Serial Lift-Out has the potential to improve throughput and contextual continuity, but these advantages must be weighed against the additional technical demands of extraction, attachment, and serial sectioning.

## Conclusion

The practical implementation of cryo-ET ultimately depends on whether target-containing lamellae can be secured consistently and whether interpretable tomograms can be produced reproducibly from those lamellae. Cryo-FIB-SEM is a central platform that goes beyond lamella fabrication to unify the entire process into a single workflow, from targeting by cryo-CLEM and failure-mode management of contamination, charging, curtaining, and devitrification to cryo-ET acquisition and interpretation. As workflows extend to thicker specimens, preparation options such as HPF and the waffle method may become effective; even then, the bottleneck shifts from the technique itself to the systematization of targeting and QC. Emerging approaches such as Serial Lift-Out further suggest that future progress will not only depend on improved instrumentation, but also on practical solutions for redeposition control, lamella stability, and scalable throughput. As the application scope of cryo-ET broadens in the future, success will depend less on better instruments and more on standardized workflows and operational capability, including stepwise decision-making and validation.

## Data Availability

Not applicable.

## Abbreviations

cryo-EM: cryo-electron microscopy
cryo-ET: cryo-electron tomography
SPA: single-particle analysis
STA: sub-tomogram averaging
FIB: focused ion beam
SEM: scanning electron microscopy
cryo-FIB-SEM: cryo–focused ion beam-scanning electron microscopy
cryo-CLEM: cryo-correlative light and electron microscopy
LM: light microscopy
TEM: transmission electron microscopy
CTF: contrast transfer function
HPF: high-pressure freezing
ROI: region of interest
QC: quality control
